# Clinical trials during pandemic in primary care: Low number and low validity after one-year experience

**DOI:** 10.1080/13814788.2021.1986279

**Published:** 2021-10-11

**Authors:** Athina Tatsioni, Iliana Siountri, Donatos Tsamoulis, Kyriaki Vafeidou

**Affiliations:** Research Unit for General Medicine and Primary Health Care, Faculty of Medicine, School of Health Science, University of Ioannina, Ioannina, Greece

During the pandemic, primary care (PC) core components, including access, comprehensiveness and continuity of services as well as person-centred care were challenged in all countries [1–2]. Numerous articles on COVID-19 and PC, including opinion papers, narrative reviews, and descriptive studies, appeared in the literature. However, well-designed trials remain crucial to reduce uncertainty in harm-benefit ratio for PC interventions and facilitate risk communication. Searching in PubMed (February 2021), we identified only 10 trials [3–12], and 1 pilot study conducted in PC (Supplementary Material) [13]. Based on these published trials, we try to delineate certain methodological issues that pertain to the components of a clinical trial, i.e. design, population, intervention, comparator and outcome. Subsequently, we suggest potential approaches that may overcome these issues in future PC trials.

Clinical trials in PC generally lacked a randomised design, and a comparator group. Notably, there were only two trials [3,4], including a control group; one of them with a randomised design (RCT) [3]. Non-controlled trials might have been informative on the feasibility of interventions. However, without a comparison group, they could hardly reach a robust conclusion on the effectiveness and safety.

In addition, PC trials usually did not focus on high-risk populations [14–18], including older patients and people with underlying health conditions. There was only one trial on nursing home residents [9]; and three trials on participants with chronic diseases [3,4,11]. Moreover, published trials did not focus on under privileged populations as defined by social determinants of health. Aggressive measures taken in most countries to mitigate COVID-19 led to reduced access, disruptive health care and significant social and economic repercussions [19–21], especially where patients struggled with the technology and had increasing mental health issues. Previous reports warned of increased risk of morbidity and mortality from deferred referrals and routine PC services [22–24].

Another methodological issue raised is the heterogeneity of evaluated interventions, with the majority designed as complex interventions. Trials recruiting patients with suspected and confirmed COVID-19 evaluated diverse interventions, including diagnostic modalities [6–8,10–13], medications, telehealth use and multidisciplinary care models without including a control group. Evaluation of multi-component interventions to deliver preventive care services for non-COVID-19 conditions was practically missing except for a non-controlled trial assessing drive-through transcutaneous bilirubin screening for newborns [5]. Previous studies documented a decline in preventive services, such as childhood immunisation coverage and cancer screening [25–27]. There was no trial on interventions to promote or sustain COVID-19 vaccination. However, relevant trials may still be in progress.

Finally, another issue that poses difficulties in the interpretation of results is the type of assessed outcomes. Trials on suspected and confirmed COVID-19 patients [6–8,10–13] measured a variety of short-term outcomes, including patient health outcomes, such as symptom control, recovery, hospitalisations and mortality as well as health services outcomes, such as monitoring, cost, time and appropriate referral. Feasibility outcomes, including home monitoring and programme changes after feedback, were also reported. Few trials on patients with chronic illness evaluated both patient health outcomes [3,4,11], i.e. depression symptom control, patient satisfaction and health services outcomes, i.e. visit attendance, level of integration of services. The only trial on preventive care focussed on feasibility and health services utilisation outcomes [5].

Several recommendations may help overcome the methodological issues described above. In the initial phase of pandemic, the design of high-quality RCTs might be a difficult task. Researchers may justifiably avoid comparisons with placebo or no treatment during a pandemic. Instead, they may design trials including a control group to facilitate comparisons of complex interventions with usual care, i.e. telehealth versus in-person care, comparisons of timing of implementation, comparisons of different sequencing of interventions and comparisons of different combinations of interventions [28]. Intent-to-treat estimations provided by RCTs are particularly relevant for interventions where adherence could be problematic or where the post-randomisation experiences of compared groups could be very different [29], i.e. when multi-component interventions introduce several associated secondary changes.

In addition, eligibility criteria should consider other determinants of health [30,31], especially social factors that may jeopardise health care equity and affect outcomes. Besides high-risk populations, future RCTs recruiting under-privileged populations could provide robust evidence on specific multi-component interventions that may reduce inequalities. They may either compare precise implementation of interventions in high-risk populations and settings versus populations with lower risk or use risk information to target the populations and settings where interventions are to be applied [28]. Both short and long-term consequences due to health care inequalities during the pandemic may become evident in the years to come. Therefore, PC should have efficient interventions in place to support resilient health care.

To address issues due to heterogeneity of complex interventions, large RCTs may come closer to real-life circumstances using a pragmatic design, especially when assessing major clinical outcomes such as hospitalisations and death. For example, well-designed RCTs with or without a telehealth component may indicate how to best provide care for patients with acute and chronic illnesses as well as for patients in need of preventive and palliative care. Collaborative and integrated health care models need to be evaluated beyond the level of proof-of-concept study. Other designs potentially suitable for large PC trials include cluster RCTs of parallel clusters or stepped-wedge RCTs [32]. During the design for RCTs, several issues may appear, such as how to consider carry-over effects from sequential treatments and interactions. Methodological recommendations based on experience from other medical fields, including crossover, adaptive and factorial RCT designs may be considered in PC [28,33]. RCTs may also assess the impact of diagnostic modalities beyond diagnostic outcomes, evaluating their impact on therapeutic choice, patient and societal outcomes [34]. In addition, they may assess the impact of newly developed and validated clinical prediction models compared to usual clinical assessment [35].

Despite the variety of outcomes, to evaluate effectiveness and safety, efforts should be made to measure outcomes that matter. PC may follow the paradigm of other medical fields (e.g. the Core Outcome Measures in Effectiveness Trials initiative http://www.comet-initiative.org/) in developing core outcome measures relevant to the care provided. Long-term outcomes are equally crucial to short-term outcomes. All trials except for the RCT by Hickey et al. [3] supported that their results encouraged further adoption. Future RCTs need to confirm replication of these results before widespread use. Besides, morbidity and mortality trials may also assess the impact of pandemic on other diseases, health care utilisation, social indicators (e.g. violence), quality of life and self-care attainment.

The very small number of published trials, most of them without a control group, indicates that robust evidence on efficient PC interventions is not available one year after the pandemic started. An updated search in September 2021 showed that a few additional RCTs evaluating mainly drug interventions for patients with COVID-19 in the community appeared in PubMed. One may argue that most studies, both RCTs and others, may have limited usefulness [36,37]. However, COVID-19 may offer PC an opportunity to use experience from research in other fields to design trials that have a maximum chance of being useful and informative.

## Supplementary Material

Supplemental Material
