# Supplementary material for: Clinical trials during pandemic in primary care: Low number and low validity after one-year experience
Source: Eur J Gen Pract. 2021 Oct 11;27(1):274–6. doi: 10.1080/13814788.2021.1986279 (PMC8510587; doi:10.1080/13814788.2021.1986279)
Supplement: Supplemental Material [file IGEN_A_1986279_SM2804.docx]

**Brief description of literature search including results**

*Purpose:*

We aimed at identifying published clinical trials assessing interventions during the pandemic in PC and critically appraise on their outcomes.

*Search strategy:*

We searched PubMed (last search February 2021) using keywords on SARS-CoV-2, and PHC. Specifically, we used the following:

(SARS-CoV2 OR COVID OR COVID19 OR COVID-19 OR pandemic) AND ("Family Practice"[Mesh] OR "Primary Health Care"[Mesh] OR "Physicians, Family"[Mesh] OR "family medicine" OR "family practice" OR "general medicine" OR "general practice" OR "general internal medicine" OR "family physician" OR "general practitioner" OR "primary care" OR "primary health care")

We excluded trials evaluating public health interventions on wider population groups.

*Eligible Trials*

We identified 4,653 unique items. After excluding 4,397 items based on title or abstract, we screened 256 in full text. Out of the 256 items, we excluded 188, which were not clinical trials; 39 as not relevant to PC; and 18 as not relevant to the pandemic. Thus, we included 10 trials [3-12] with a total of 8,891 participants and one pilot study [13] with 37 patients. The characteristics of the 11 trials are shown in the Table. The duration of the eligible trials ranged from 6 days to 4 months.

**Table.** Characteristics of eligible trials

| **First author, publication year; country** | **Sample size; study design** | **Population** | **Intervention** | **Outcome** |
| --- | --- | --- | --- | --- |
| Hickey, 2020, USA [3] | 201; RCT | HIV positive patients | Structured pre-visit planning call (telehealth)* | Telemedicine visit attendance |
| Garcia-Huidobro, 2020; Chile [4] | 5149; CCT | Patients with chronic illness in a large private academic health network | Telehealth use** | Patient satisfaction |
| Chua, 2020; Malaysia [5] | 1842; NCCT | New-borns | Drive-through transcutaneous bilirubin screening | Babies who needed venesection or phototherapy; duration; cost; crowding; reported issues with the technique |
| Judson, 2020; USA [6] | 950; NCCT | Suspected COVID-19 patients | Telehealth use for self-triage and self-scheduling tool | Time to schedule a visit |
| Blazey-Martin, 2020; USA [7] | 305; NCCT | COVID-19 patients | Telehealth use | Patients who recovered/ admitted/ admitted and discharged/ died; program changes after feedback |
| Yen, 2020; Taiwan [8] | 217; NCCT | Suspected COVID-19 patients: quarantined travellers | Telehealth use | Infection monitoring; cost |
| Salas-Coronas, 2020; Spain [9] | 48; NCCT | COVID-19 patients: nursing home residents | Multidisciplinary care model | Mortality; hospitalizations |
| Calvo‐Cebrián, 2020; Spain [10] | 61; NCCT | Suspected COVID-19 patients | Lung ultrasound | Appropriate referral |
| Birch, 2021; USA [11] | 58; NCCT | Patients diagnosed with depression | Collaborative care model | Level of integration; depression symptom control |
| Della-Torre, Italy; 2020 [12] | 9; NCCT | COVID-19 patients | Colchicine | Symptom control |
| Banzi, 2020; Italy [13] | 37; NCCT  Pilot study | COVID-19 and suspected COVID-19 patients | Home monitoring | Appropriate hospital referral |

RCT, randomized controlled trial; CCT, non-randomized controlled clinical trial; NCCT, non-controlled clinical trial

*Comparator: standard reminder

**Comparator: in person care
